# Efficacy and mortality of rotating sheaths versus laser sheaths for transvenous lead extraction: a meta-analysis

**DOI:** 10.1007/s10840-021-01076-x

**Published:** 2021-11-27

**Authors:** Sun Yong Lee, Isabel E. Allen, Celso Diaz, Xiaofan Guo, Cara Pellegrini, Ramin Beygui, Ricardo Cardona-Guarache, Gregory M. Marcus, Byron K. Lee

**Affiliations:** 1 Division of Cardiology, University of California, San Francisco, San Francisco, CA 94143 USA; 2 Department of Internal Medicine, San Joaquin General Hospital, 500 W Hospital Rd, French Camp, CA 95231 USA; 3 Department of Epidemiology and Biostatistics, University of California, San Francisco, San Francisco, CA 94143 USA; 4 School of Medicine, University of California, San Francisco, San Francisco, CA 94143 USA; 5 Department of Medicine, Northwestern University, Chicago, IL 60611 USA; 6 Department of Cardiology, The First Hospital of China Medical University, Shenyang, Liaoning, China; 7 Division of Cardiology, San Francisco VAMC, San Francisco, CA 94121 USA; 8 Division of Adult Cardiothoracic Surgery, San Francisco, CA 94143 USA; 9 Department of Heart and Vascular (Cardiology), Clinical Cardiac Electrophysiology, The Everett Clinic, Everett, WA 98201 USA

**Keywords:** Transvenous lead extraction, Laser sheaths, Rotating sheaths, CIEDs, Mortality

## Abstract

**Background:**

Rotating and laser sheaths are both routinely used in transvenous lead extraction (TLE) which can lead to catastrophic complications including death. The efficacy and risk of each approach are uncertain. To perform a meta-analysis to compare success and mortality rates associated with rotating and laser sheaths.

**Methods:**

We searched electronic academic databases for case series of consecutive patients and randomized controlled trials published 1998–2017 describing the use of rotating and laser sheaths for TLE. Among 48 studies identified, rotating sheaths included 1,094 patients with 1,955 leads in 14 studies, and laser sheaths included 7,775 patients with 12,339 leads in 34 studies. Patients receiving rotating sheaths were older (63 versus 60 years old) and were more often male (74% versus 72%); CRT-P/Ds were more commonly extracted using rotating sheaths (12% versus 7%), whereas ICDs were less common (37% versus 42%), *p* > 0.05 for all. Infection as an indication for lead extraction was higher in the rotating sheath group (59.8% versus 52.9%, *p* = 0.002). The mean time from initial lead implantation was 7.2 years for rotating sheaths and 6.3 years for laser sheaths (*p* > 0.05).

**Results:**

Success rates for complete removal of transvenous leads were 95.1% in rotating sheaths and 93.4% in laser sheaths (*p* < 0.05). There was one death among 1,094 patients (0.09%) in rotating sheaths and 66 deaths among 7,775 patients (0.85%) in laser sheaths, translating to a 9.3-fold higher risk of death with laser sheaths (95% CI 1.3 to 66.9, *p* = 0.01).

**Conclusions:**

Laser sheaths were associated with lower complete lead removal rate and a 9.3-fold higher risk of death.

**Supplementary Information:**

The online version contains supplementary material available at 10.1007/s10840-021-01076-x.

## Introduction

The rate of cardiac implantable electronic device (CIED) implantation and life expectancy has increased worldwide over recent years [1, 2]. As a result, the need for transvenous lead extraction (TLE) has also increased. TLE is a challenging procedure as there are risks of fibrous adhesions between the leads, vascular wall, and endocardial surface. These structures can tear during TLE which can lead serious complications such as cardiovascular injury and death. In order to prevent these complications, advanced procedures are commonly used [1, 2]. The two common approaches to TLE utilize either laser sheaths or rotating sheaths [3]. To disrupt the fibrotic attachments of indwelling leads, laser sheaths employ fiber-optics to transmit desiccating laser light while rotating sheaths utilize a revolving bladed distal tip [1, 3, 4]. Although, technology improvements have increased efficacy and safety, TLE procedures can still lead to serious, life-threatening complications including death [1, 3, 5]. Laser sheaths are more commonly utilized world-wide, setting the stage for a familiarity or even allegiance bias among operators. However, there are now several case series describing the outcome of patients who undergo TLE with either the laser sheath or the rotating sheath published in the peer-reviewed literature, but no large studies comparing these two approaches directly [6]. We therefore performed a meta-analysis to summarize these data in hopes of providing clinically relevant estimates based on the peer-reviewed literature regarding the relative efficacy and safety of these two approaches.

## Methods

We systematically searched PubMed, Embase in September 2017 using the following medical subject heading terms: *transvenous lead extraction*, *mechanical dilator sheath*, *Evolution mechanical dilator sheath*, *laser sheaths*, *Excimer laser*, *pacemaker*, *defibrillator*. Two authors (L. SY, L. BK) independently extracted the data after predefined search criteria.

Studies were included in this meta-analysis if they met all the following eligibility criteria:

1. Case series of consecutive patients or randomized controlled trials (RCTs)
2. Reporting more than ten patients and including a minimum set of data: number of subjects undergoing TLE and number of extracted leads, mean age of the patients, lead age, type of device, success rate, and number of deaths
3. Written in English

Studies were excluded if they were:

1. Systematic reviews
2. Letters
3. Point-of-views or editorials

If the same center(s) produced different publications with duplicate cases reported due to time window overlapping, only the study with the highest number of patients (usually the one published latest) was included. If more than one group of patients was described in the same study, the groups were handled as if they were from two separate studies.

Complete procedural success was defined as the removal of all targeted leads and all lead material from the vascular space, with the absence of any permanently disabling complication or procedure related death [1, 6, 7]. Clinical success was defined as the removal of all targeted leads and lead material from the vascular space, or retention of a small portion of the lead that does not negatively impact the outcome goals of the procedure. Failure was defined as inability to achieve either complete procedural or clinical success, or the development of any permanently disabling complication or procedure-related death. Any permanently disabling complication included cardiac avulsion or tear requiring surgical interventions, vascular avulsion or tear requiring surgical interventions, pulmonary embolism requiring surgical interventions, or stroke.

The main goal of the analysis was to compare the efficacy and safety of laser sheaths and rotating sheaths. Several stratified meta-analyses were performed to find the risk of bias including center volume, length of lead age, device type, and publication year [2]. For center volume, Lexicon [2, 8] criteria were used based on the number of procedures performed over 4 years per site as indicated in Online Supplemental Table S1. In order to provide a more objective analysis, we focused on procedure-related death and complete procedure success rate. Major or minor complications were not considered since the definitions of these complications were variable among studies. We also analyzed TLE indication in the two procedure groups and we restricted to studies published 2009 or later in both procedures to reduce temporal bias.

We included in the meta-analysis only the studies on laser sheaths and rotating sheaths for TLE procedures. Since almost all included studies were observational case series except two RCTs, no traditional meta-analysis of head-to-head comparisons was possible [2, 9]. We therefore used meta-analyses of proportions to combine data from each case series to get summary estimates of the absolute risk of each safety outcome and the Meta-analysis Of Observational Studies in Epidemiology (MOOSE) statement was applied [10]. Summary estimates were produced using random effects meta-analyses to control for heterogeneity between studies. Sensitivity analyses were examined using meta-regression and subgroup analyses. Baseline characteristics of the studies used means (standard deviations) for continuous variables and counts (percents) for categorical data. Comparison between groups used Student’s *t*-test for continuous variables and chi-squared tests for categorical variables. All analyses used Stata 15.1 (College Station, TX).

## Results

**Fig. 1:**
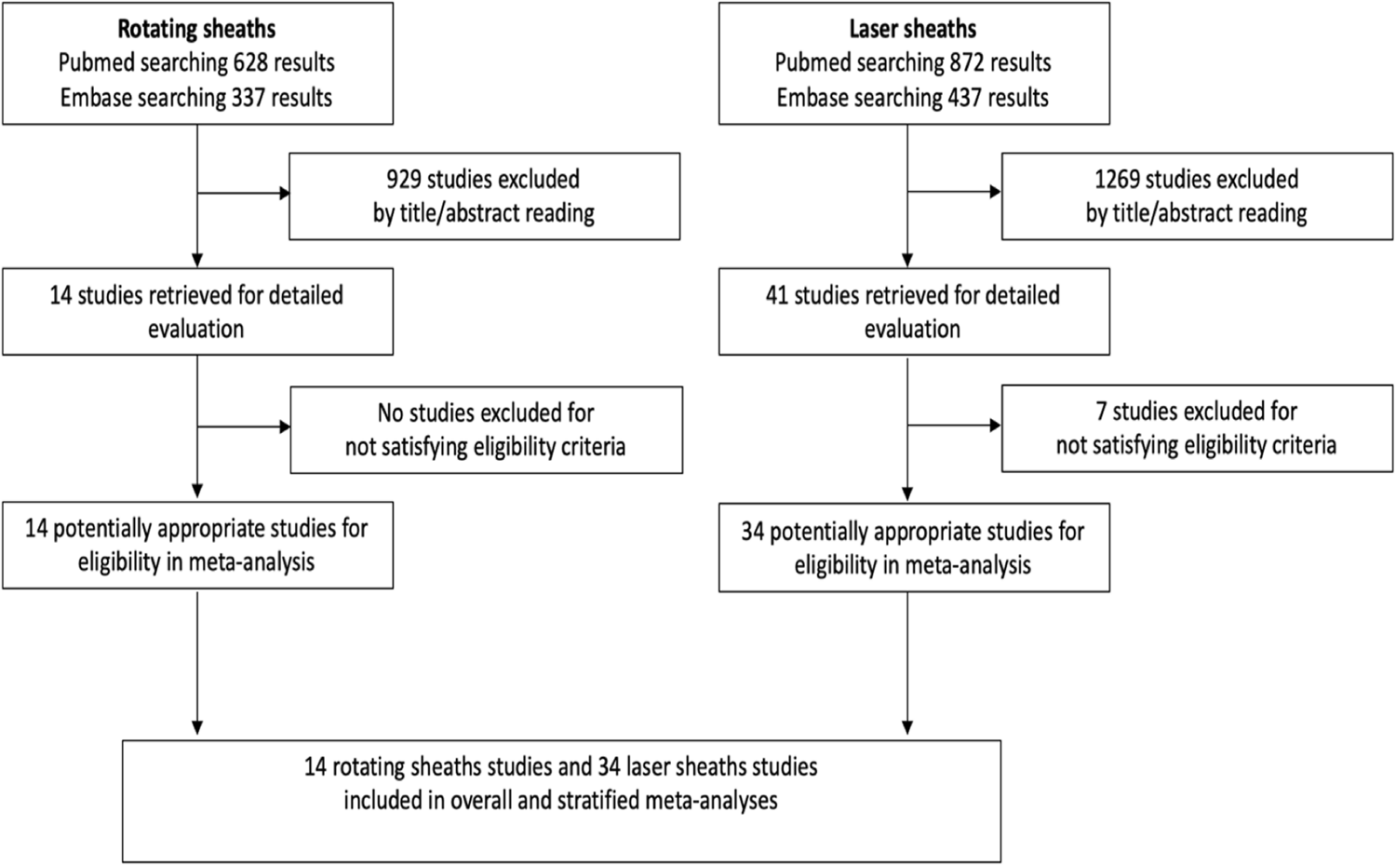
Flow of the included studies in each stage of the bibliographic search as per PRISMA checklist

The literature search for randomized trials yielded 55 studies in PubMed and an additional 3 in Embase. Two of these randomized trials included laser sheaths. In the next-step, a broader search was conducted after deleting “randomized” and adding procedure-specific terms (Fig. 1). The literature search on rotating sheaths yielded 628 studies by PubMed and 337 studies by Embase. On review of the titles and abstracts, 929 studies were excluded as being unrelated to the field of research or clearly identifying a type of manuscript in the systematic review: reviews, letters, editorials, and case reports of ≤ 10 subjects. Total 14 studies on rotating sheaths were included for meta-analysis which met eligibility criteria.

Eight hundred seventy-two studies were found in PubMed and 437 studies in Embase on laser sheaths. A total of 1269 studies were excluded as being unrelated to the field of research or clearly identifying a type of manuscript included in the systematic review: reviews, letters, editorials, and case reports of ≤ 10 subjects. Seven out of 41 studies on laser sheaths were further excluded for not satisfying eligibility criteria. A total of 34 studies on laser sheaths were included for meta-analysis.

In total, 48 rotating and laser sheath studies met eligibility criteria and were included in the meta-analysis: 2 were RCTs [11, 12]; 3 were prospective observational studies [7, 13, 14]; and 43 had a retrospective observational design [6, 8, 11, 15–53].

The 48 identified studies described 8,869 patients who underwent attempted extraction of 14,294 leads with rotating sheaths or laser sheaths (Table 1, Online Supplemental Table S2-3) [5–8, 11–54]. The patients undergoing TLE with rotating sheaths were older and were more often male. Within the rotating sheath group, CRT-P/Ds were more commonly extracted, whereas ICDs were less common than the laser sheath group (*p* > 0.05 for all). Complete procedural success per lead was achieved in approximately 95% of cases utilizing a rotating sheath (pooled estimate of 11/ 14 studies) and 93% of cases utilizing a laser sheath (pooled estimate of 21/34 studies) [22].

**Table 1:** Baseline characteristics of two procedures

		Rotating sheaths	Laser sheaths	*P*	*RR*
Age (years, mean ± SD)		62.68 ± 3.906	59.78 ± 12.72	0.44	-
Male (% ± SD)		73.70 ± 8.442	71.74 ± 9.651	0.54	-
Patient number		1,094	7,775	-	-
Number of leads		1,955	12,339	-	-
Lead age (years, mean ± SD)		7.164 ± 1.441	6.278 ± 2.173	0.17	-
Implanted device	% of PM (% ± SD)	52.88 ± 18.17	46.86 ± 31.88	0.58	-
	% of ICD (% ± SD)	36.76 ± 15.69	41.73 ± 30.77	0.59	-
	% of CRT-P/D (% ± SD)	12.17 ± 16.53	7.267 ± 12.77	0.35	-
Indications for TLE	Infective	495 (59.8%)	3,291 (52.9%)	0.0002	-
	Non-infective	333 (40.2%)	2,932 (47.1%)	-	-
Complete procedural success rate per leads (%)		95.1317	93.3936	0.0069	RR = 1.357
Clinical success rate (% ± SD)		99.18 ± 1.043	96.72 ± 6.245	0.19	-
Death rate (%)		1/1094 (0.09)	66/7775 (0.85)	0.011	RR = 9.287

*P*, *p* value; *RR*, relative risk; *SD*, standard deviation; *CI*, confidence interval; *PM*, pacemaker; *ICD* implantable cardioverter defibrillator; *CRT-P/D*, cardiac resynchronization therapy pacemaker/defibrillator

The total number of procedure-related deaths in both groups was 67: one out of 1,094 patients (0.09%) in the rotating sheath group and 66 out of 7,775 patients (0.85%) in the laser sheath group, translating to a 9.3-fold higher risk of death with laser sheaths (95% CI 1.3 to 66.9, *p* = 0.01). The causes of deaths for patients who underwent rotating or laser sheath lead extraction are shown in Table 2. The one patient death associated with a rotating sheath resulted from cardiovascular injury [17]. The portion of deaths due to cardiovascular injury following laser sheath procedures was 39.4% (26/66). The superior vena cava (SVC) was the most common site of injury accounting for over 30% of cases (Table 3).

**Table 2:** Cause of death for patients who underwent rotating or laser sheath lead extraction

	Rotating sheath extraction *N* (%)	Laser sheath extraction *N* (%)
Confirmed/suspected Cardiovascular Injury	1 (100)	26 (39.4)
Pulmonary embolism	-	2 (3.0)
Arrhythmia	-	2 (3.0)
Infection	-	27 (40.9)
Sepsis	-	3 (4.5)
Endocarditis	-	1 (1.5)
Endocarditis or pocket infection	-	23 (34.8)
Renal failure	-	2 (3.0)
Unknown	-	7 (10.6)

**Table 3:** Cardiovascular structures injured during the procedure amongst patients that died during rotating or laser sheath lead extraction

	Rotating sheath extraction *N* (%)	Laser sheath extraction *N* (%)
Unknown	1 (100)	12 (46.2)
Superior vena cava	-	7 (26.9)
Right ventricle	-	2 (7.7)
Right atrium	-	1 (3.8)
Anomalous innominate arteriovenous fistula	-	1 (3.8)
Subclavian vein	-	1 (3.8)
Superior vena cava to right atrium	-	1 (3.8)
Inferior vena cava to right atrium	-	1 (3.8)

We analyzed TLE indication in the two groups (Table 4). Infection was more often an indication for extraction in the rotating sheath group at 59.8% compared to the laser group at 52.9%, *p* = 0.0002.

**Table 4:** Analysis of TLE indications

	Rotating sheath patient number (%)	Laser sheath patient number (%)
Endocarditis, pocket infection, or systemic infection	495 (59.8%)	3,291 (52.9%)
Lead malfunction	215 (26.0%)	1,647 (26.5%)
Lead displacement	2 (0.2%)	445 (7.2%)
Upgrade the device	91 (11.0%)	109 (1.8%)
Chronic pain	2 (0.2%)	47 (0.8%)
Venous thrombosis	1 (0.1%)	84 (1.3%)
Other	22 (2.7%)	600 (9.6%)
Total	828	6,223

Indications were analyzed when data available

When we limited analyses to medium- and high-volume centers by using hospital-volume Lexicon study criteria, complete procedural lead removal was achieved in 95% of cases in the rotating sheath group and 93% of cases in the laser sheath group (*p* < 0.05) [2, 8, 55]. Among medium- and high-volume centers, the mortality rate was 0% (0/841) in the rotating sheath group and 0.87% (62/7094) in laser sheath group (*p* = 0.01).

When we restricted analyses to years 2009–2017 to control for temporal bias since rotating sheaths were introduced in 2009, complete procedural lead removal rate was 95.1% in the rotating sheath group and 96.7% in the laser sheath group (*p* < 0.05). The total death number was 1 out of 1,094 patients in the rotating group (0.09%) and 41 out of 4,313 patients (0.95%) in the laser group, translating to a 10.4-fold higher risk of death with laser sheaths (95% CI 1.4 to 75.6, *p* = 0.004).

The results of separate analyses for RCTs and observational case series studies are presented in Online Supplemental Tables S4-S8. In one of the two RCTs, information was limited in allocation concealment, selective outcome reporting, and blinding of participants and personnel [11]. All included studies showed low or unclear risk of bias for all other domains. Forty-six case series studies presented a score of four to eight of the eight points according to the scale adopted by NICE.

As shown in Table 1 and Online Supplemental Tables S2-3, studies were heterogeneous in that they had various numbers of enrolled populations and different hospital volumes, and there are fewer studies (publication study from 2009) for rotating sheaths being a newer device. The meta-analysis of 48 studies showed a high heterogeneity between the studies for the complete procedural success rate (test for heterogeneity 250.60, degree of freedom = 28, *p* < 0.009, *I*^^2^ = 88.83%). The heterogeneity for the death rate (test for heterogeneity 54.26, degree of freedom = 45, *p* = 0.16, *I*^^2^ = 17.06%) was not highly variable.

In a meta-regression analysis, our study showed that the lead age and percent of ICD leads did not prove to be significantly associated with any of the outcomes among the various subgroups. The detailed analysis is available in supplemental materials with Online Supplemental Figures S1-S6.

## Discussion

The primary finding of this meta-analysis of 48 studies involving 8,869 patients is that laser sheaths are associated with a lower complete lead removal rate (93% vs. 95% for rotating sheath, *p* < 0.05) and a 9.3-fold higher risk of death (95% CI 1.3 to 66.9, *p* = 0.01). The difference in mortality remained unchanged when the analysis is limited to studies from medium- and high-volume centers or studies performed 2009 or later. These mortality results are consistent with a recently published study based on mortality data obtained from the Manufacturer and User Facility Device Experience (MAUDE) database which found lead removal using laser sheaths to be associated with approximately 7 times greater risk of death when compared to rotating sheaths (95% CI 4.1 to 12.7, *p* < 0.0001) [56].

Although low-volume centers have been found to have a higher rate of death and complications compared to high-volume centers, this did not account for our findings [2]. The comparison of complete lead removal rate remained similar when limiting analysis to studies from medium- and high-volume centers (95% vs. 93% for laser sheath, *p* < 0.05) and mortality was still higher with laser sheaths (0% vs. 0.87% for laser sheath).

Since the rotating sheaths were introduced over 10 years after the laser sheath, we were concerned that temporal bias could be playing a role in our findings. However, limiting analysis of complete lead removal to studies starting in 2009 (when the first rotating sheath study was published) found similar complete removal rates (96.7% vs. 95.1% for rotating sheath) and still a 10.4-fold higher risk of death (95% CI 1.4 to 75.6, *p* = 0.004) in laser sheath.

As we expected, one of the main causes of death from TLE is cardiovascular injuries. SVC is the most commonly injured structure (Table 3). The mechanism of SVC injury is unclear. Although the penetration of the laser sheath has been reported to be shallow, repeated activation of the laser sheath at a site of heavy fibrosis may lead to buildup of thermal injury and, as a result, cause vascular injuries. SVC injury may be less likely with rotating sheaths because the cutting tip is only 1.27 mm thick (Cook Evolution). Therefore, only tissue very near the sheath tip (and lead body) can be affected [12].

The ancillary analysis of the ELECTRa (European Lead Extraction ConTRolled) study supports our findings [57]. They looked at the major cardiac and vascular complications after TLE and also found that rotating sheaths had lower incidence of complication compared to laser sheaths. The number of CV major complications, 49 among 3,510 patients who underwent TLE, such as vascular avulsion or tear (VA/T) due to SVC laceration and cardiac avulsion or tear (CA/T) with tamponade was higher with laser sheaths 19.06% compared to rotating sheaths 7.61% (*p* < 0.002). Both CA/T and VA/T requiring pericardiocentesis/chest tube/surgical procedure (p/c/s) were higher in laser sheaths than in rotating sheaths. However, mortality rate with each sheath type is not reported.

It is unclear why laser sheaths were associated with more non-cardiovascular deaths, which included deaths due to pulmonary embolism, arrhythmia, infection, renal failure, and unknown causes. We speculate that some of these deaths were due to unrecognized cardiovascular injury, perhaps in cases where there was no autopsy. Additionally, the heat generated by the laser may promote clotting, embolization, electrical ectopy, or vegetation dissolution that may also lead to some of these non-cardiovascular deaths.

A new intravenous occlusion balloon designed to seal accidental tears in the SVC during TLE was introduced in July 2016 by Spectranetics Corporation (Colorado Springs, CO) [58]. However, this device was recalled due to the possibility of a blocked guidewire lumen in some units which would delay life-saving treatment and may result in immediate and serious adverse consequences including death. Our study was unable to assess the impact of the intravenous occlusion balloon since many of the studies analyzed were from before July 2016 and few of the later studies disclosed its availability. Although widespread of availability of this device may decrease mortality rates associated with both rotating and laser sheaths, it would not prevent the initial occurrence of an SVC tear which is a serious complication to be avoided.

Another factor that may influence success rate and mortality is the indication for lead removal. The LexICon study showed that patients with infection (as an indication for lead removal) tend to have worse short-term prognosis [8]. Therefore, we analyzed indications for procedure to see if there were any imbalance among patient groups as there was a concern that patients who underwent laser sheath TLE might be sicker and less stable. In both groups, there were no significant differences in TLE indications (Table 4). Interestingly, the rotating sheath group had a slightly higher proportion of infection (59.8% vs 52.9% for laser sheath) despite of a lower death rate. Therefore, infection cannot explain the higher risk of mortality seen with laser sheaths.

Our results are similar to those reported in a smaller meta-analysis that reviewed different TLE approaches including simple traction, classic mechanical, laser sheaths, and rotating sheaths. This study demonstrated that the risk of death or major complication was higher with the use of laser sheaths when compared to mechanical methods [4].

Our meta-regression studies showed that the study year, lead age, and hospital volume do not affect the death rate comparison. There was a higher death rate among pacemaker patients in both laser and rotating sheath groups. This finding contrasts a previous observation that dual coiled ICD lead is an independent risk factor predicting major complications [4]. Our meta-regression analysis also showed that the complete procedure success rate improved over time with the progression of study years in both laser and rotating sheath procedures. This finding is in line with the results of a study by Ghosh et al., which can be attributed to a learning effect [52]. When patients were divided into three consecutive groups by study years, complete success rate was higher in the latter third. However, as noted above, limiting analysis to studies starting in 2009 (when the first rotating sheath study was published) or later found similar complete removal rates with the two sheath types, and still a much higher risk of death with laser sheaths.

Recognizing that conclusions from meta-analyses are not definitive, we consider our result hypothesis generating and warranting further research. A randomized controlled trial comparing laser sheaths to rotating sheaths would be valuable.

## Study limitations

There are several limitations of our meta-analysis and meta-regression analysis. First, our study was not based on randomized-controlled trials like traditional meta-analyses. Instead, our analyses relied mainly on case series studies which can potentially introduce selection bias. There was also high heterogeneity among the total sets of selected studies. We made several efforts to minimize these possible biases. We confirmed that both procedures were performed in groups with similar indications. Subgroup analysis sorted by hospital volume and study year yielded conclusions similar to our main findings.

Second, there were technological advances in both groups which could have affected the results. During the study time frame, for laser sheaths, there was the introduction of the 80-Hz laser which replaced the 40-Hz laser. For rotating sheaths, there was the introduction of the Cook Medical bidirectional rotating sheath which replaced their unidirectional rotating sheath, and Spectranetics introduced the TightRail rotating sheath in 2014. In general, studies did not indicate the sub-types of laser sheath or rotating cutting sheath used and therefore, its effect on the results could not be determined.

Finally, crossovers to the other sheath type during TLE could have also affected the results. However, we reviewed the studies and there was no mention of crossover noted in rotating sheath studies. In contrast, a few of the laser studies noted crossover. In Fu et al., one of the studies in the laser sheath group, they used a rotating sheath when laser extraction was not successful. However, they analyze the groups separately and the major complication rate was 0% with crossover to rotating sheaths and 3.7% in laser only extraction procedures [27]. In Starck et al., they allowed the crossover from mechanical to laser or vice versa, yet crossover never occurred [33]. Therefore, the chance of bias due to crossover is low.

## Conclusion

Despite greater comorbidities in those undergoing extraction using rotating sheaths, our meta-analysis found that laser sheaths were associated with lower complete lead removal rate and a 9.3-fold higher risk of death.

## Supplementary Information

ESM 1 (DOCX 1.20 MB)

## Abbreviations

TLE: Transvenous lead extraction
CIEDs: Cardiac implantable electronic devices
RCTs: Randomized controlled trials
EHRA: European Heart Rhythm Association
NICE: National Institute for Clinical Excellence
PM: Pacemaker
ICD: Implantable cardioverter defibrillator
CRTP/D: Cardiac resynchronization therapy pacemaker/defibrillator
ES: Effect size
CI: Confidence interval
SVC: Superior vena cava
RA: Right atrium
RV: Right ventricle
CA/T: Cardiac avulsion or tear
VA/T: Vascular avulsion or tear
P/C/S: Pericardiocentesis/chest tube/surgical procedure
