# Supplementary material for: Efficacy and mortality of rotating sheaths versus laser sheaths for transvenous lead extraction: a meta-analysis
Source: J Interv Card Electrophysiol. 2021 Nov 27;66(5):1067–75. doi: 10.1007/s10840-021-01076-x (PMC10333355; doi:10.1007/s10840-021-01076-x)
Supplement: Supplementary file 1 — (DOCX 1.20 MB) [file 10840_2021_1076_MOESM1_ESM.docx]

**SUPPLEMENTAL MATERIAL**

**Supplemental Methods**

**European Heart Rhythm Association (EHRA) and Lexicon criteria for lead extractor centers**

For center volume, Lexicon criteria were used based on the number of procedures performed over four years per site as indicated in Online Supplemental Table S1.(1, 2)

Being aware of the potential bias associated with analysis of 46 case series studies, we performed a quality assessment of the included studies with a methodological tool previously used to assess the quality of case series for a set of clinical guidelines issued by the National Institute for Clinical Excellence (NICE) and in a similar meta-analysis. (3-5) Two reviewers (L. SY, L. BK) independently scored each study using a form (Online Tables S2-S6) and where disparities occurred, the final judgment was performed with a third author (A. IE).

**Quality assessment**

There were two RCTs and 46 case series among the analyzed 48 studies. One study described multiple cohorts that were analyzed as separate case series. (6) Although the Cochrane Collaboration’s tool was used for assessing risk of bias in RCTs, a comprehensive review of this risk was not practical because the comparative groups in these two RCTs did not exclusively involve rotating or laser sheaths. (6-8) Being aware of the potential bias associated with analysis of 46 case series studies, we performed a quality assessment of the included studies with a methodological tool previously used to assess the quality of case series for a set of clinical guidelines issued by the National Institute for Clinical Excellence (NICE) and in a similar meta-analysis. (3-5) Two reviewers (L. SY, L. BK) independently scored each study using a form (Online Supplemental Tables S2-S6) and where disparities occurred, the final judgment was performed with a third author (A. IE).

**Systematic review registration to PROSPERO**

This study was registered to PROSPERO which is an international database of prospectively registered systematic reviews in health and social care, welfare, public health, education, crime, justice, and international development, where there is a health-related outcome. PROSPERO is funded by the National Institute for Health Research and produced by CRD, which is an academic department of the University of York. Our registration number is CRD42017081076.

**Supplemental Table S1** - European Heart Rhythm Association (EHRA) and Lexicon criteria for lead extractor centers

**Supplemental Table S2**. Rotating sheaths studies with baseline characteristics

**Supplemental Table S3**. Laser sheaths studies with baseline characteristics

**Supplemental Table S4 -** Quality assessment of included case series in rotating sheaths

**Supplemental Table S5 -** Quality assessment of included case series in laser sheaths

**Supplemental Table S6 -** Quality assessment of included case series in laser sheaths

**Supplemental Table S7-** Quality assessment of included case series in laser sheaths

**Supplemental Table S8 -** Quality assessment of included prospective randomized controlled studies

**Supplemental Table S1**. European Heart Rhythm Association (EHRA) and Lexicon criteria for lead extractor centers

| **EHRA criteria for lead extractor centers** |
| --- |
| Low volume center <15 procedures/year  Non-training center 15-30 procedures/year  Training center >30 procedures/year |
| **Lexicon study criteria for extractor centers** |
| Low volume center <60 procedures over 4 years period  Medium volume center 60-130 procedures over 4 years period  High volume center >130 procedures over 4 years period |

**Supplemental Table S2**. Rotating sheaths studies with baseline characteristics

| **First author, Study year (Ref. #)** | **Country** | **Design** | **Multi-centric** | **Patient number** | **EHRA criteria^*^** | **Lexicon criteria** **^†^** | **Mean age** | **Male (%)** | **Number of leads** | **Lead age (years)** | **% of PM** | **% of ICD** | **% of CRT-P/D** | **Complete procedural success rate (%)**  **Per leads** | **Clinical success rate (%)** | **Death number (%)** |
| --- | --- | --- | --- | --- | --- | --- | --- | --- | --- | --- | --- | --- | --- | --- | --- | --- |
| Mazzone, Patrizio et al., 2017 (9) | Italy | Prospect | Yes | 124 | >30 | 60-130 | 65 | 84.7 | 238 | 7.7 | NA | 38.2 | NA | 235/238 (98.7%) | 100 | 0 |
| Domenichini. Giulia. Et al., 2017 (10) | UK | Retros | No | 212 | 15-30 | <60 | 65.9 | 67.9 | 389 | 6.2 | 39.6 | 39.2 | 21.2 | 375/389 (96.4%) | NA | 0 |
| Aytemir, Kudret et al., 2016 (11) | Turkey | Observ | No | 23 | >30 | >130 | 59.1 | 82.6 | 42 | 6 | 43.4 | 30.4 | 26 | 41/42 (97.6%) | 100 | 0 |
| Kocabas, Ugur. et al., 2016 (12) | Turkey | Retros | No | 41 | <15 | <60 | 61.5 | 73.2 | 67 | 7.4 | 65.8 | 34.2 | 0 | NA | 97.5 | 1 (2.4%) |
| Starck, Christoph T. et al., 2016 (13) | Germany, Switzerland | Retros | Yes | 40 | 15-30 | 60-130 | NA | NA | 52 | 8.4 | 46.2 | 53.8 | 0 | 46/52 (88.5%) | 98.1 | 0 |
| Delnoy, Peter Paul H.M., 2016 (14) | The Netherlands | Prosp | No | 77 | 15-30 | 60-130 | 66 | 71 | 111 | 8.0 | NA | 19 | NA | 98/111 (88%) | 98.1 | 0 |
| Kong, Jihua. Et al., 2015 (15) | China | Retros | No | 17 | 15-30 | 60-130 | 67 | 58.8 | 31 | 10.8 | 88.2 | 5.9 | 5.9 | NA | 100 | 0 |
| Sharma, S. et al., 2015 (16) | USA | Retros | No | 262 | >30 | >130 | NA | NA | 438 | 5.7 | NA | 70 | NA | 416/438(95%) | 100 | 0 |
| Mazzone, Patrizio. Et al., 2013 (17) | Italy | Retros | No | 48 | 15-30 | 60-130 | 65.4 | 77.1 | 81 | 8.4 | 23.4 | 27.7 | 48.9 | NA | 97.9 | 0 |
| Oto, A. et al., 2012 (18) | Turkey | Retros | No | 66 | >30 | >130 | 55.6 | 66.6 | 140 | 7.1 | 42.3 | 39.4 | 19.7 | 133/140 (95%) | 98.5 | 0 |
| Aksu, Tolga. Et al., 2012 (19) | Turkey | Retros | No | 12 | 15-30 | 60-130 | 58 | 83 | 14 | 6.1 | 67 | 33 | 0 | 13/14 (92.9%) | 100 | 0 |
| Oto, A. et al., 2011 (20) | Turkey | Retros | No | 23 | 15-30 | 60-130 | 58.6 | 78 | 41 | 6.2 | 60.9 | 39.1 | 0 | 35/41 (85.4%) | 100 | 0 |
| Hussein, Ayman A. et al., 2010 (21) | USA, Lebanon | Retros | No | 29 | 15-30 | 60-130 | 64.4 | 79 | 41 | 5.4 | 52 | 48 | 0 | 33/41 (80.5%) | 100 | 0 |
| Kutarski. Andrzej. et al., 2009 (22) | Poland | Retros | No | 120 | >30 | 60-130 | 65.7 | 62.5 | 270 | 6.9 | NA | NA | NA | 236/270 (87.4%) | NA | 0 |
| Prosp = prospective study; Retros = retrospective study; Observ = observational study; PM = pacemaker; ICD = implantable cardioverter defibrillator; CRTP/D = cardiac resynchronization therapy pacemaker/defibrillator.  ^*^EHRA Criteria: European Heart Rhythm Association Criteria. See Table 1  **^†^**Lexicon Criteria: See Table 1  NA, not available. | | | | | | | | | | | | | | | | |

**Supplemental Table S3**. Laser sheaths studies with baseline characteristics

| **First author, Study year (Ref. #)** | **Country** | **Design** | **Multi-centric** | **Patient number** | **EHRA criteria^*^** | **Lexicon criteria^†^** | **Mean age** | **Male (%)** | **Number of leads** | **Lead age (years)** | **% of PM** | **% of ICD** | **% of CRT-P/D** | **Complete procedural success rate (%)**  **Per leads** | **Clinical success rate (%)** | **Death number (%)** |
| --- | --- | --- | --- | --- | --- | --- | --- | --- | --- | --- | --- | --- | --- | --- | --- | --- |
| Pecha. Simon. Et al., 2017 (23) | Germany | Retros | No | 151 | >30 | >130 | 66.2 | 73.5 | 292 | 8.2 | 27.0 | 27.4 | NA | 285/292 (97.6%) | 99.3 | 0 |
| Pecha, Simon et al., 2016 (24) | Germany | Retros | No | 171 | <15 | <60 | 58.2 | 70.8 | 186 | 3.9 | 0 | 100 | 0 | 182/186 (97.8%) | 98.2 | 0 |
| Williams, Kenneth J. et al., 2016 (25) | Canada | Retros | No | 108 | <15 | <60 | 67.2 | 73.1 | 218 | 7.5 | 70.5 | 23.0 | 6.7 | 203/218 (93.1%) | 97.2 | 1 (0.93) |
| Okamura. Hideo. Et al., 2016 (26) | Japan | Retros | No | 40 | 15-30 | 60-130 | 65.5 | 65 | 70 | 7.3 | 62.5 | 27.5 | 9 | 68/70 (97.1%) | 100 | 0 |
| Fu, Hai-Xia. Et al., 2015 (27) | US | Retros | No | 331 | 15-30 | 60-130 | 63 | 71 | 601 | 8.3 | 39 | 38 | 23 | NA | NA | 2 (0.60) |
| Hakmi, Samer et al., 2014 (28) | Germany | Retros | No | 38 | >30 | >130 | 62.0 | 73.7 | 76 | 8.0 | 48.7 | 51.3 | 0 | 72/76 (94.8%) | 94.7 | 0 |
| Tanawuttiwat. Tanyanan. Et Al., 2014 (29) | US | Retros | No | 427 | >30 | >130 | 67.9 | 72.6 | 821 | 5.71 | 34.2 | 42.2 | 23.6 | 765/821 (93.2%) | NA | 1 (0.23) |
| Sohal, Manav. Et al., 2014 (30) | UK | Retros | No | 71 | <15 | <60 | 62 | 77 | 129 | 6.7 | 27 | 37 | 36 | NA | NA | 0 |
| Wang, Wei. et al., 2014 (31) | Canada | Retros | No | 140 | 15-30 | 60-130 | 62.6 | 78.6 | 279 | 7.9 | 62.9 | 37.1 | 0 | NA | NA | 1 (0.71) |
| Starck, Christoph T. et al., 2013 (32) | Switzerland | Retros | No | 39 | <15 | <60 | NA | NA | NA | 6.9 | 30.8 | 69.2 | 0 | NA | 76.9 | 0 |
| Patel, Divyang. Et al., 2013 (33) | US | Retros | No | 18 | <15 | <60 | 57.1 | 83.3 | 18 | 4.0 | 0 | 100 | 0 | 17/18 (94.4%) | NA | 0 |
| Maytin, Melanie. Et al., 2013 (34) | US | Retros | Yes | 12 | <15 | <60 | 45 | 50 | 20 | 6.2 | 0 | 100 | 0 | 20/20 (100%) | 100 | 0 |
| Mazzone, Patrizio. Et al., 2013 (17) | Italy | Retros | No | 73 | 15-30 | 60-130 | 60.2 | 79.5 | 127 | 5.2 | 17.8 | 34.2 | 47.9 | NA | 98.6 | 0 |
| Maytin, Melanie. Et al., 2011 (35) | US | Retros | No | 46 | <15 | <60 | 58 | 63 | 51 | 1.4 | 13 | 74 | 13 | NA | 100 | 0 |
| Rodriguez, Yasser. Et al., 2011 (36) | US | Retros | No | 506 | >30 | >130 | 69.1 | 74.9 | 1067 | 3.6 | 41.3 | 42.7 | 16.0 | 1067/1067(100%) | NA | 1 (0.20) |
| Bordachar. Pierre. Et al., 2010 (6) | France | Prospective randomized | No | 50 | >30 | >130 | 69 | 76.0 | 115 | 12 | 88 | 12 | 0 | NA | NA | 0 |
|  | France | Nonrandomized | Yes | 218 | >30 | >130 | 71 | 77.1 | 458 | 9 | 83 | 17 | 0 | NA | NA | 2 (0.92) |
| Kratz, John M. et al., 2010 (37) | US | Retros | No | 270 | 15-30 | 60-130 | NA | NA | NA | NA | NA | NA | 0 | NA | NA | 3 (1.11) |
| Wazni, Oussama et al., 2010 (2) | US, Canada | Observational retros | Yes | 1449 | >30 | >130 | 63.4 | 71.8 | 2405 | 6.8 | 70 | 29.2 | NA | 2322/2405 (96.5%) | 97.7 | 27 (1.86) |
| Gaca. Jeffrey G. et al., 2009 (38) | US | Retros | No | 112 | 15-30 | 60-130 | 59.8 | 72 | 205 | 5.8 | NA | NA | NA | NA | NA | 3 (2.68) |
| Scott, Paul A. et al., 2009 (39) | UK | Retros | No | 43 | <15 | <60 | 66 | 67 | 80 | 8.8 | 60 | 19 | 21 | NA | NA | 0 |
| Roux, Jean-Francois. Et al., 2007 (40) | Canada | Prosp | No | 175 | >30 | >130 | 62 | 74 | 270 | 7.8 | NA | 18 | NA | 241/270 (89.3%) | NA | 1 (0.57) |
| Kennergren, C. et al., 2007 (41) | Sweden, UK, Germany, Switzerland, The Netherlands, Portugal, Denmark, Belgium | Retros | Yes | 292 | >30 | >130 | 61.6 | 69.9 | 383 | 6.2 | NA | 13.1 | NA | 348/383 (90.9%) | NA | 0 |
| Moak, Jeffrey P. et al., 2006 (42) | US | Retros | No | 25 | NA | NA | 13.9 | NA | 43 | 4.1 | 83.7 | 16.3 | 0 | 39/43 (91%) | NA | 0 |
| Ghosh, Nina, et al., 2005 (43) | Canada | Retros | No | 75 | 15-30 | 60-130 | 63 | 78.7 | 145 | 8.5 | 90 | 10 | 0 | 139/145 (95.9%) | NA | 0 |
| Bracke, F.A.L.E. et al., 2004 (44) | The Netherlands | Retros | No | 55 | NA | NA | NA | NA | NA | NA | NA | NA | 0 | NA | NA | 2 (3.64) |
| Cooper, Joshua M. et al., 2003 (45) | US | Retros | No | 14 | <15 | <60 | 17.9 | NA | 20 | 3.5 | 19.0 | 81.0 | 0 | 19/20 (95%) | NA | 0 |
| Byrd, Charles L. et al., 2002 (46) | US | Retros | Yes | 1684 | >30 | >130 | 64 | 64 | 2561 | 6.3 | NA | NA | NA | 2305/2561 (90%) | NA | 13 (0.77) |
| Costa. Roberto. Et al., 2001 (47) | Brazil | Retros | No | 36 | 15-30 | 60-130 | 54.2 | 61.1 | 56 | 7.5 | 83.9 | 16.1 | 0 | 46/56 (82.1%) | NA | 0 |
| Gilligan, D.M. et al., 2001 (48) | US | Prosp | No | 34 | <15 | <60 | 64.0 | 100 | 50 | 5.0 | 66 | 33 | 0 | 48/50 (96%) | 100 | 0 |
| Epstein. Laurence M. et al., 1999 (49) | US | Retros | Yes | 863 | >30 | >130 | 63.4 | 63.0 | 1285 | 6.4 | NA | NA | 0 | 1133/1285 (88.2%) | 91-92% | 7 (0.81) |
| Wilkoff, Bruce L et al., 1999 (8) | US | Randomized | Yes | 153 | >30 | >130 | 65 | 67 | 244 | 5.4 | 100 | 0 | 0 | 230/244 (94%) | 94.8 | 1 (0.65) |
| Kennergren, Charles. Et al., 1998 (50) | Sweden | Retros | No | 45 | >30 | >130 | 65.1 | 51.1 | 50 | 4.0 | NA | NA | NA | NA | NA | 0 |
| Krishnan, Subramanian C. et al., 1998 (51) | US | Retros | No | 11 | NA | NA | 65.9 | 81.8 | 14 | 3.0 | 0 | 100 | 0 | 14/14(100%) | 100 | 1 (9.09) |
| Prosp = prospective study; Prospective randomized = prospective randomized controlled trial; Nonrandomized = nonrandomized observational study; Observational retros = observational retrospective study; Retros = retrospective study; Observ = observational study; Randomized = randomized controlled trial; PM = pacemaker; ICD = implantable cardioverter defibrillator; CRTP/D = cardiac resynchronization therapy pacemaker/defibrillator.  ^*^EHRA Criteria: European Heart Rhythm Association Criteria. See Table 1  ^†^Lexicon Criteria: See Table 1  NA, not available. | | | | | | | | | | | | | | | | |

**Supplemental Table S4 -** Quality assessment of included case series in rotating sheaths

| Criteria (Yes = 1, No = 0) | Study | | | | | | | | | | | | | |
| --- | --- | --- | --- | --- | --- | --- | --- | --- | --- | --- | --- | --- | --- | --- |
|  | Mazzone (2017) | Domen-  ichini | Aytemir | Kocabas | Starck | Delnoy | Kong | Sharma | Mazzone (2013) | Oto (2012) | Aksu | Oto (2011) | Hussein | Kutarski |
| Case series from more than one centre? | 1 | 0 | 0 | 0 | 1 | 0 | 0 | 0 | 0 | 0 | 0 | 0 | 0 | 0 |
| Aim of study clearly described? | 1 | 1 | 1 | 1 | 1 | 1 | 1 | 1 | 1 | 1 | 1 | 1 | 1 | 1 |
| Case definition clearly reported? | 1 | 1 | 1 | 1 | 0 | 0 | 0 | 0 | 1 | 0 | 0 | 0 | 1 | 1 |
| Clear definition of outcomes reported? | 1 | 1 | 1 | 1 | 1 | 1 | 1 | 0 | 1 | 1 | 1 | 1 | 1 | 0 |
| Data collected prospectively? | 1 | 0 | 0 | 0 | 0 | 1 | 0 | 0 | 0 | 0 | 0 | 0 | 0 | 0 |
| Patients recruited consecutively? | 1 | 1 | 1 | 1 | 1 | 1 | 1 | 1 | 1 | 1 | 1 | 1 | 1 | 1 |
| Main findings clearly described? | 1 | 1 | 1 | 1 | 1 | 1 | 1 | 1 | 1 | 1 | 1 | 1 | 1 | 1 |
| Are outcomes stratified? | 1 | 1 | 1 | 1 | 1 | 1 | 1 | 1 | 1 | 1 | 1 | 1 | 1 | 1 |
| Total (out of 8) | 8 | 6 | 6 | 6 | 6 | 6 | 5 | 4 | 6 | 5 | 5 | 5 | 6 | 5 |

**Supplemental Table S5 -** Quality assessment of included case series in laser sheaths

| Criteria (Yes = 1, No = 0) | Study | | | | | | | | | | |
| --- | --- | --- | --- | --- | --- | --- | --- | --- | --- | --- | --- |
|  | Pecha (2017) | Pecha (2016) | Williams | Fu | Hakmi | Tanawuttiwat | Sohal | Wang | Okamura | Starck | Patel |
| Case series from more than one centre? | 0 | 0 | 0 | 0 | 0 | 0 | 0 | 0 | 0 | 0 | 0 |
| Aim of study clearly described? | 1 | 1 | 1 | 1 | 1 | 1 | 1 | 1 | 1 | 1 | 1 |
| Case definition clearly reported? | 0 | 0 | 1 | 1 | 0 | 1 | 1 | 0 | 0 | 0 | 1 |
| Clear definition of outcomes reported? | 1 | 1 | 1 | 1 | 1 | 1 | 0 | 0 | 1 | 1 | 0 |
| Data collected prospectively? | 0 | 0 | 0 | 0 | 0 | 0 | 0 | 0 | 0 | 0 | 0 |
| Patients recruited consecutively? | 1 | 1 | 1 | 1 | 1 | 1 | 1 | 1 | 1 | 1 | 1 |
| Main findings clearly described? | 1 | 1 | 1 | 1 | 1 | 1 | 1 | 1 | 1 | 1 | 1 |
| Are outcomes stratified? | 1 | 1 | 1 | 1 | 1 | 1 | 1 | 1 | 1 | 1 | 1 |
| Total (out of 8) | 5 | 5 | 6 | 6 | 5 | 6 | 5 | 4 | 5 | 5 | 5 |

**Supplemental Table S6 -** Quality assessment of included case series in laser sheaths

| Criteria (Yes = 1, No = 0) | Study | | | | | | | | | | |
| --- | --- | --- | --- | --- | --- | --- | --- | --- | --- | --- | --- |
|  | Maytin (2013) | Mazzone (2013) | Maytin (2011) | Rodriguez | Bordachar | Kratz | Wazni | Gaca | Scott | Roux | Kennergren (2007) |
| Case series from more than one centre? | 1 | 0 | 0 | 0 | 1 | 0 | 1 | 0 | 0 | 0 | 1 |
| Aim of study clearly described? | 1 | 1 | 1 | 1 | 1 | 1 | 1 | 1 | 1 | 1 | 1 |
| Case definition clearly reported? | 0 | 1 | 1 | 1 | 1 | 0 | 1 | 0 | 0 | 0 | 0 |
| Clear definition of outcomes reported? | 1 | 1 | 1 | 1 | 1 | 0 | 1 | 1 | 1 | 1 | 1 |
| Data collected prospectively? | 0 | 0 | 0 | 0 | 1 | 0 | 1 | 0 | 0 | 1 | 0 |
| Patients recruited consecutively? | 1 | 1 | 1 | 1 | 1 | 1 | 1 | 1 | 1 | 1 | 1 |
| Main findings clearly described? | 1 | 1 | 1 | 1 | 1 | 1 | 1 | 1 | 1 | 1 | 1 |
| Are outcomes stratified? | 1 | 1 | 1 | 1 | 1 | 1 | 1 | 1 | 1 | 1 | 1 |
| Total (out of 8) | 6 | 6 | 6 | 6 | 8 | 4 | 8 | 5 | 5 | 6 | 6 |

**Supplemental Table S7 -** Quality assessment of included case series in laser sheaths

| Criteria (Yes = 1, No = 0) | Study | | | | | | | | | |
| --- | --- | --- | --- | --- | --- | --- | --- | --- | --- | --- |
|  | Moak | Ghosh | Bracke | Cooper | Byrd | Costa | Gilligan | Epstein | Kennergren (1998) | Krishnan |
| Case series from more than one centre? | 0 | 0 | 0 | 0 | 1 | 0 | 0 | 1 | 0 | 0 |
| Aim of study clearly described? | 1 | 1 | 1 | 1 | 1 | 1 | 1 | 1 | 1 | 1 |
| Case definition clearly reported? | 0 | 0 | 0 | 0 | 1 | 0 | 0 | 1 | 0 | 0 |
| Clear definition of outcomes reported? | 0 | 1 | 0 | 0 | 1 | 0 | 0 | 1 | 0 | 0 |
| Data collected prospectively? | 0 | 0 | 0 | 0 | 0 | 0 | 1 | 0 | 0 | 0 |
| Patients recruited consecutively? | 1 | 1 | 1 | 1 | 1 | 1 | 1 | 1 | 1 | 1 |
| Main findings clearly described? | 1 | 1 | 1 | 1 | 1 | 1 | 1 | 1 | 1 | 1 |
| Are outcomes stratified? | 1 | 1 | 1 | 1 | 1 | 1 | 1 | 1 | 1 | 1 |
| Total (out of 8) | 4 | 5 | 4 | 4 | 7 | 4 | 5 | 7 | 4 | 4 |

**Supplemental Table S8 -** Quality assessment of included prospective randomized controlled studies

| Study | Adequate random sequence generation | Allocation concealment | Selective outcome reporting | Blinding of participants and personnel | Blinding of outcome assessment | Incomplete outcome data | Other biases |
| --- | --- | --- | --- | --- | --- | --- | --- |
| Bordachar | Low | Unclear | Unclear | Unclear | Low | Low | Unclear |
| Wilkoff | Low | Low | Low | Unclear | Low | Low | Unclear |

The meta-regression analysis for complete procedural success rate revealed that none of the variables were significant and as such, could not explain the heterogeneity (Online Supplemental Figure S1-S3). However, while trying to reduce heterogeneity, the meta-regression provided an interesting finding; the complete lead extraction success rate increased by year which may explain part of the heterogeneity and learning effect, although it was not statistically significant. Online Supplemental Figure S3 is a funnel plot for laser sheaths and rotating sheaths which shows that studies with low variability have similar effect sizes for complete procedural success.

The meta-regression analysis in death rate revealed a potential factor affecting procedure outcomes in death rate: percent of pacemaker devices (p=0.002) (Online Supplemental Figure S4). Two other factors approached statistical significance in death rate among the two subgroups: hospital volume (p=0.078), and study year (p=0.084) (Online Supplemental Figure S5 & S6). When the percent of pacemaker lead extraction increases, the death rate also increased in both subgroups. In terms of hospital volumes, the death rate increased in the laser sheath groups as the hospital volume increased. However, in the rotating sheath group, the death rate decreased as the hospital volume increased. According to study year, the death rate of laser sheaths has a tendency to slightly decrease as time goes by. The death rate tendency of rotating sheath procedures is difficult to determine, since there was only one death reported throughout all studies.

**Supplemental Figure S1** - Forest plot of overall success rate analysis

**Supplemental Figure S2** - Forest plot of death rate analysis

**Supplemental Figure S3** - Funnel plot for laser sheaths and rotating sheaths shows low bias and that studies with low variability have similar effect sizes for complete procedural success

**Supplemental Figure S4** - Meta-regression analysis of percent pacemaker extraction among the laser sheaths and rotating sheaths procedures in relation to the death rate

**Supplemental Figure S5** - Meta-regression analysis of hospital volume among the laser sheaths and rotating sheaths procedures in relation to the death rate

**Supplemental Figure S6** - Meta-regression analysis of study year among the laser sheaths and rotating sheaths procedures in relation to the death rate

**Supplemental Figure S1**. Forest plot of overall success rate analysis. The horizontal bars indicate 95% confidence intervals (CI) and the diamond indicates the overall success rate in the study. The grey box around each study represents the relative sample size of the study.

Dotted line refers to the pooled estimate of all the case series analyzed and subgroups by Laser and Rotating Cutting Sheath are given following those studies.

ES = effect size or death rate; CI = confidence interval

**Supplemental Figure S2.** Forest plot of death rate analysis. The horizontal bars indicate 95% confidence intervals (CI) and the diamond indicates the overall death rate in the study (most studies had no deaths). The grey box around each study represents the relative sample size of the study.

Dotted line refers to the pooled estimate of all the case series analyzed and subgroups by Laser and Rotating Cutting Sheath are given following those studies.

ES = effect size or death rate; CI = confidence interval


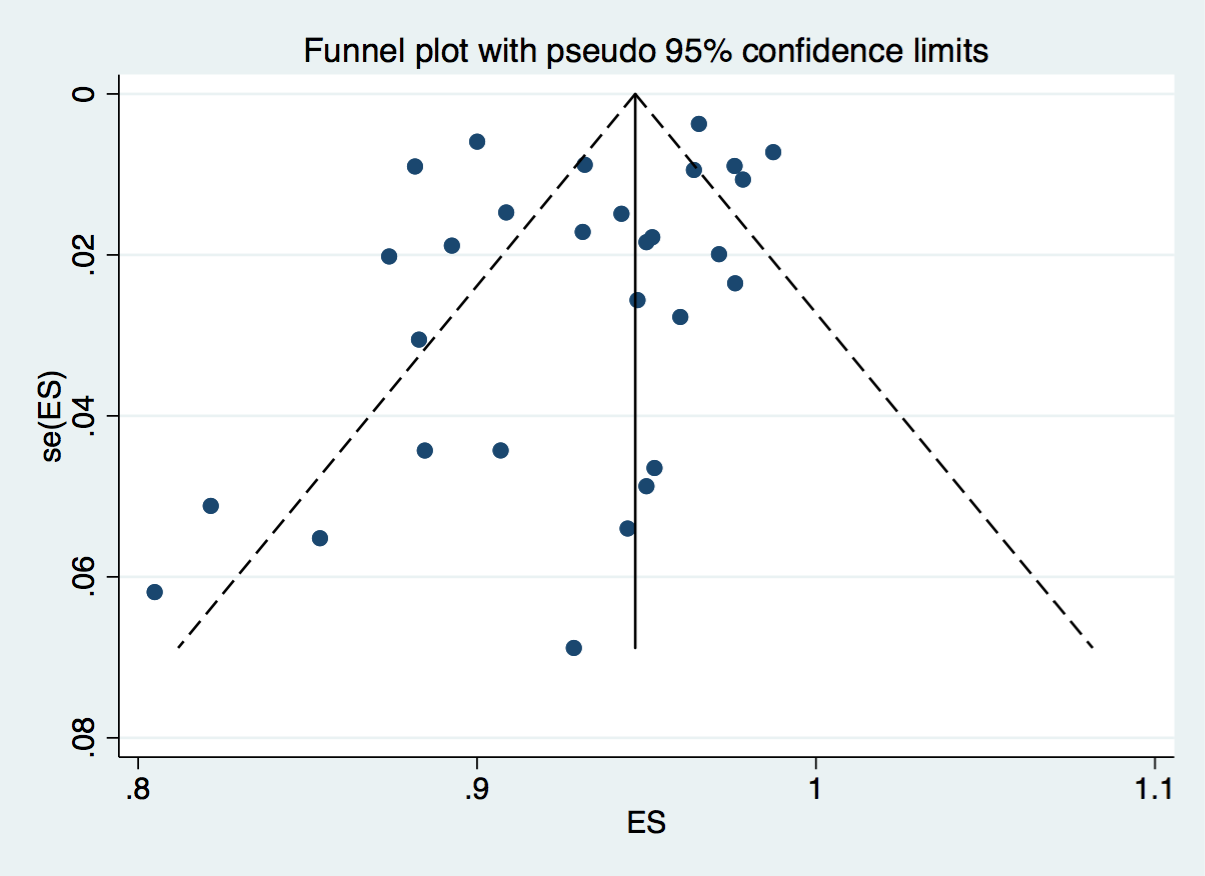


**Supplemental Figure S3**. Funnel plot for laser sheaths and rotating sheaths shows low bias and that studies with low variability have similar effect sizes for complete procedural success.


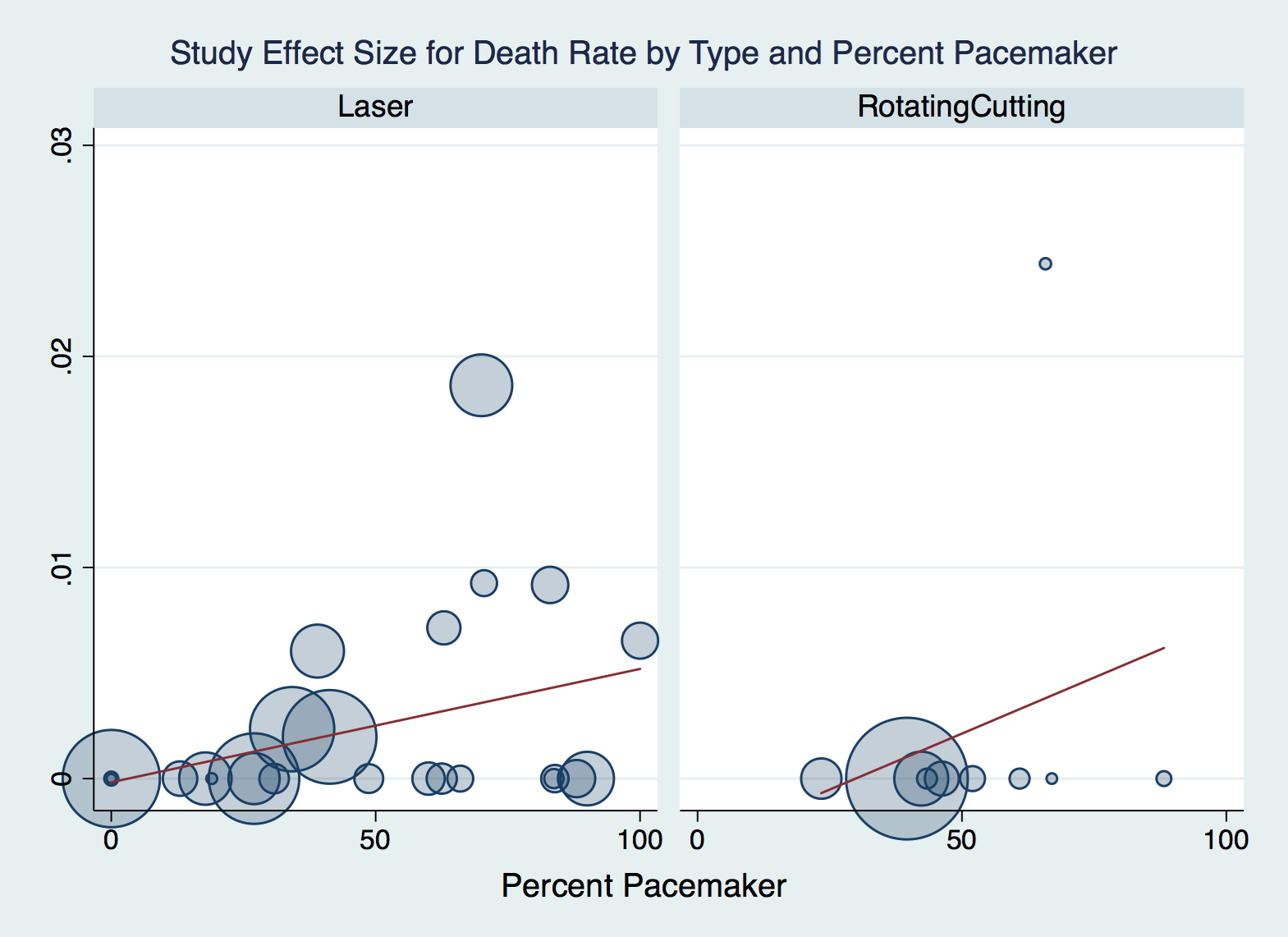


**Supplemental Figure S4**. Meta-regression analysis of percent pacemaker extraction among the laser sheaths and rotating sheaths procedures in relation to the death rate. The size of the circles corresponds to the random-effects weighting for each study.


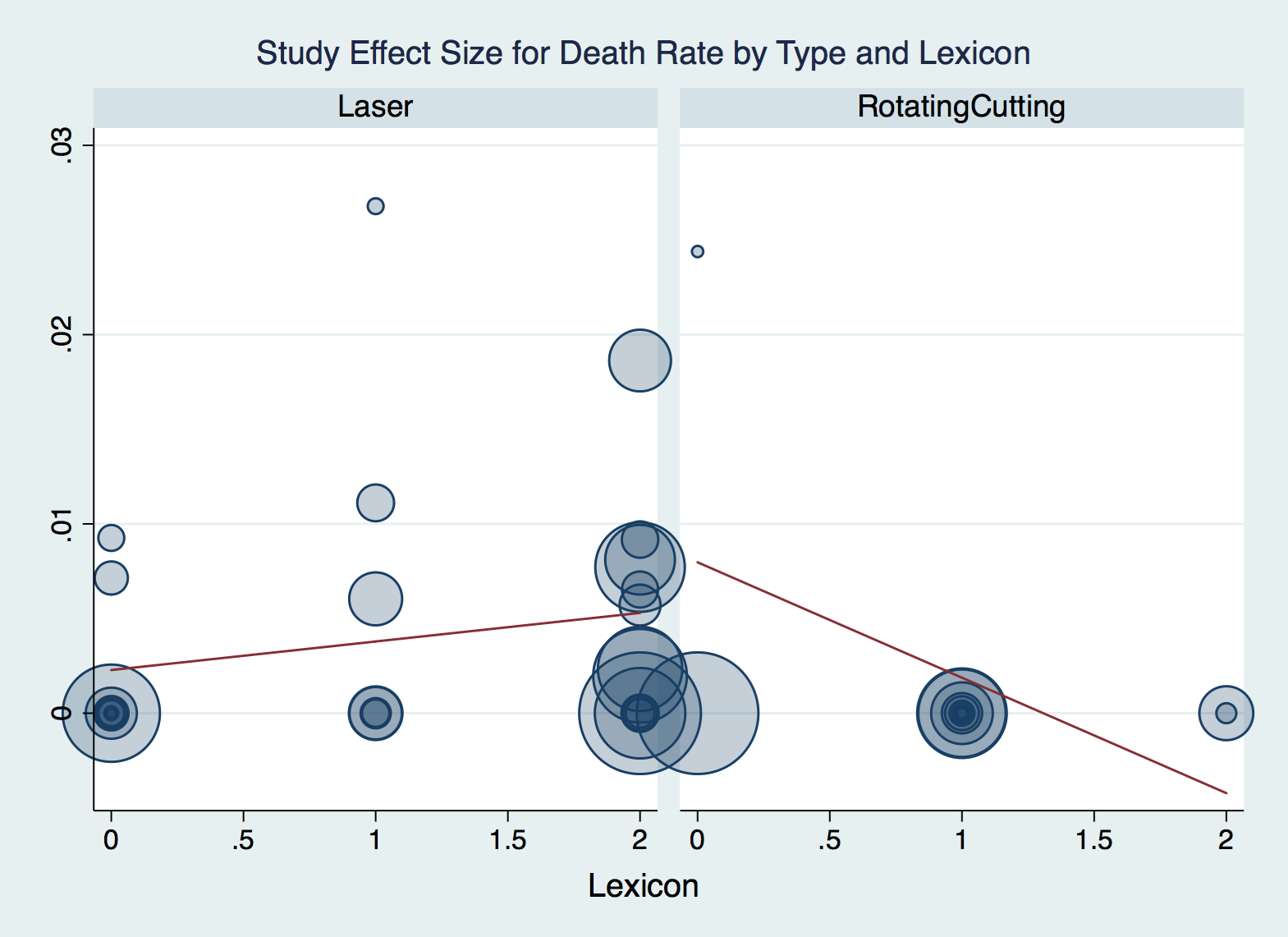


**Supplemental Figure S5.** Meta-regression analysis of hospital volume among the laser sheaths and rotating sheaths procedures in relation to the death rate. The size of the circles corresponds to the random-effects weighting for each study.


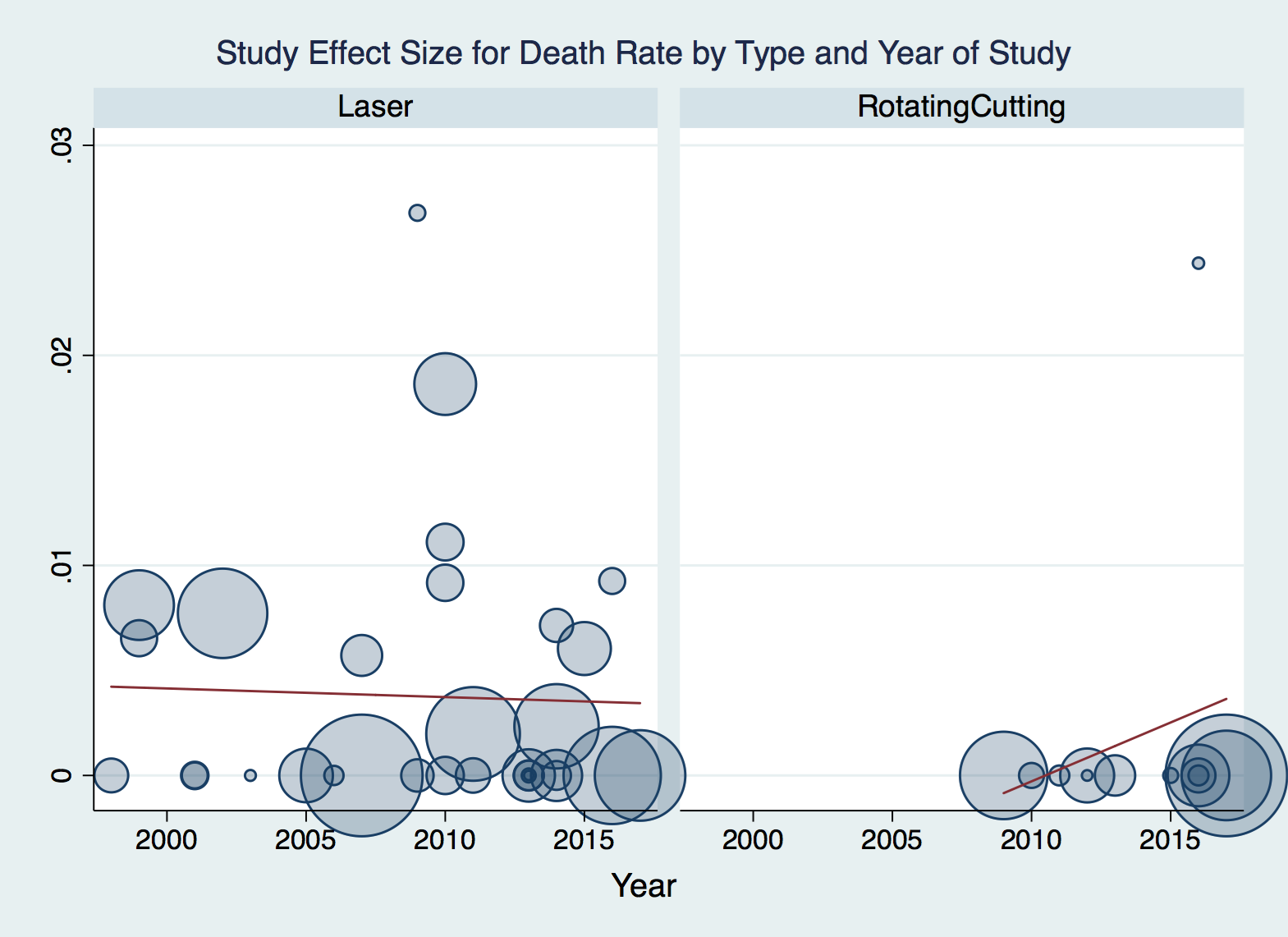


**Supplemental Figure S6**. Meta-regression analysis of study year among the laser sheaths and rotating sheaths procedures in relation to the death rate. The size of the circles corresponds to the random-effects weighting for each study.

**Supplemental References**

1. Di Monaco A, Pelargonio G, Narducci ML, Manzoli L, Boccia S, Flacco ME, et al. Safety of transvenous lead extraction according to centre volume: a systematic review and meta-analysis. Europace. 2014;16(10):1496-507.

2. Wazni O, Epstein LM, Carrillo RG, Love C, Adler SW, Riggio DW, et al. Lead extraction in the contemporary setting: the LExICon study: an observational retrospective study of consecutive laser lead extractions. J Am Coll Cardiol. 2010;55(6):579-86.

3. Diemberger I, Mazzotti A, Giulia MB, Cristian M, Matteo M, Letizia ZM, et al. From lead management to implanted patient management: systematic review and meta-analysis of the last 15 years of experience in lead extraction. Expert Rev Med Devices. 2013;10(4):551-73.

4. Reynolds TM, National Institute for H, Clinical E, Clinical Scince Reviews Committee of the Association for Clinical B. National Institute for Health and Clinical Excellence guidelines on preoperative tests: the use of routine preoperative tests for elective surgery. Ann Clin Biochem. 2006;43(Pt 1):13-6.

5. Weller MD, Nankivell PC, McConkey C, Paleri V, Mehanna HM. The risk and interval to malignancy of patients with laryngeal dysplasia; a systematic review of case series and meta-analysis. Clin Otolaryngol. 2010;35(5):364-72.

6. Bordachar P, Defaye P, Peyrouse E, Boveda S, Mokrani B, Marquie C, et al. Extraction of old pacemaker or cardioverter-defibrillator leads by laser sheath versus femoral approach. Circ Arrhythm Electrophysiol. 2010;3(4):319-23.

7. Higgins JP, Altman DG, Gotzsche PC, Juni P, Moher D, Oxman AD, et al. The Cochrane Collaboration's tool for assessing risk of bias in randomised trials. BMJ. 2011;343:d5928.

8. Wilkoff BL, Byrd CL, Love CJ, Hayes DL, Sellers TD, Schaerf R, et al. Pacemaker lead extraction with the laser sheath: results of the pacing lead extraction with the excimer sheath (PLEXES) trial. J Am Coll Cardiol. 1999;33(6):1671-6.

9. Mazzone P, Migliore F, Bertaglia E, Facchin D, Daleffe E, Calzolari V, et al. Safety and efficacy of the new bidirectional rotational Evolution(R) mechanical lead extraction sheath: results from a multicentre Italian registry. Europace. 2017.

10. Domenichini G, Gonna H, Sharma R, Conti S, Fiorista L, Jones S, et al. Non-laser percutaneous extraction of pacemaker and defibrillation leads: a decade of progress. Europace. 2017;19(9):1521-6.

11. Aytemir K, Yorgun H, Canpolat U, Sahiner ML, Kaya EB, Evranos B, et al. Initial experience with the TightRail Rotating Mechanical Dilator Sheath for transvenous lead extraction. Europace. 2016;18(7):1043-8.

12. Kocabas U, Duygu H, Eren NK, Akyildiz ZI, Ozyildirim S, Tuluce SY, et al. Transvenous extraction of pacemaker and implantable cardioverter defibrillator leads using Evolution(R) mechanical dilator sheath: a single center confirmatory experience. Springerplus. 2016;5:356.

13. Starck CT, Steffel J, Caliskan E, Holubec T, Schoenrath F, Maisano F, et al. Clinical performance of a new bidirectional rotational mechanical lead extraction sheath. Europace. 2016;18(2):253-6.

14. Delnoy PP, Witte OA, Adiyaman A, Ghani A, Smit JJ, Ramdat Misier AR, et al. Lead extractions: the Zwolle experience with the Evolution mechanical sheath. Europace. 2016;18(5):762-6.

15. Kong J, Tian Y, Guo F, Ze F, Duan J, Wang L, et al. Snare sheath versus evolution sheath in transvenous lead extraction. Int J Clin Exp Med. 2015;8(11):21975-80.

16. Sharma S, Raman AS, Hematpour K, Venkataraman R, Hariharan R. Safety and efficacy of the cook medical evolution® mechanical lead extraction system: A single center experience. Heart Rhythm. 2015;12(5):S253.

17. Mazzone P, Tsiachris D, Marzi A, Ciconte G, Paglino G, Sora N, et al. Advanced techniques for chronic lead extraction: heading from the laser towards the evolution system. Europace. 2013;15(12):1771-6.

18. Oto A, Aytemir K, Canpolat U, Yorgun H, Sahiner L, Kaya EB, et al. Evolution in transvenous extraction of pacemaker and implantable cardioverter defibrillator leads using a mechanical dilator sheath. Pacing Clin Electrophysiol. 2012;35(7):834-40.

19. Aksu T, Guray U, Sen T, Durukan M, Guray Y, Demirkan B, et al. Use of the mechanical dilator sheath for removal of endocardial leads: a single center experience. Pacing Clin Electrophysiol. 2012;35(5):514-8.

20. Oto A, Aytemir K, Yorgun H, Canpolat U, Kaya EB, Kabakci G, et al. Percutaneous extraction of cardiac pacemaker and implantable cardioverter defibrillator leads with evolution mechanical dilator sheath: a single-centre experience. Europace. 2011;13(4):543-7.

21. Hussein AA, Wilkoff BL, Martin DO, Karim S, Kanj M, Callahan T, et al. Initial experience with the Evolution mechanical dilator sheath for lead extraction: safety and efficacy. Heart Rhythm. 2010;7(7):870-3.

22. Kutarski A, Malecka B, Rucinski P, Zabek A. Percutaneous extraction of endocardial leads--a single centre experience in 120 patients. Kardiol Pol. 2009;67(2):149-56; discussion 57-8.

23. Pecha S, Linder M, Gosau N, Castro L, Vogler J, Willems S, et al. Lead extraction with high frequency laser sheaths: a single-centre experience. Eur J Cardiothorac Surg. 2017;51(5):902-5.

24. Pecha S, Yildirim Y, Gosau N, Aydin MA, Willems S, Treede H, et al. Laser lead extraction allows for safe and effective removal of single- and dual-coil implantable cardioverter defibrillator leads: A single-centre experience over 12 years. Interact Cardiovasc Thorac Surg. 2017;24(1):77-81.

25. Williams KJ, O'Keefe S, Legare JF. Creation of the sole regional laser lead extraction program serving Atlantic Canada: initial experience. Can J Surg. 2016;59(3):180-7.

26. Okamura H. Lead extraction using a laser system: Techniques, efficacy, and limitations. J Arrhythm. 2016;32(4):279-82.

27. Fu HX, Huang XM, Zhong LI, Osborn MJ, Asirvatham SJ, Espinosa RE, et al. Outcomes and Complications of Lead Removal: Can We Establish a Risk Stratification Schema for a Collaborative and Effective Approach? Pacing Clin Electrophysiol. 2015;38(12):1439-47.

28. Hakmi S, Pecha S, Sill B, Reiter B, Willems S, Aydin MA, et al. Initial experience of pacemaker and implantable cardioverter defibrillator lead extraction with the new GlideLight 80 Hz laser sheaths. Interact Cardiovasc Thorac Surg. 2014;18(1):56-60.

29. Tanawuttiwat T, Gallego D, Carrillo RG. Lead extraction experience with high frequency excimer laser. Pacing Clin Electrophysiol. 2014;37(9):1120-8.

30. Sohal M, Williams S, Akhtar M, Shah A, Chen Z, Wright M, et al. Laser lead extraction to facilitate cardiac implantable electronic device upgrade and revision in the presence of central venous obstruction. Europace. 2014;16(1):81-7.

31. Wang W, Wang X, Modry D, Wang S. Cardiopulmonary bypass standby avoids fatality due to vascular laceration in laser-assisted lead extraction. J Card Surg. 2014;29(2):274-8.

32. Starck CT, Rodriguez H, Hurlimann D, Grunenfelder J, Steffel J, Salzberg SP, et al. Transvenous lead extractions: comparison of laser vs. mechanical approach. Europace. 2013;15(11):1636-41.

33. Patel D, Adelstein E, Nemec J, Mendenhall GS, Bazaz R, Jain S, et al. Extraction of defibrillator leads recalled for cable externalization and failure. J Interv Card Electrophysiol. 2013;36(3):273-8.

34. Maytin M, Henrikson CA, Schaerf RH, Epstein LM, John RM. Multicenter experience with transvenous lead extraction in arrhythmogenic right ventricular cardiomyopathy (ARVC). Pacing Clin Electrophysiol. 2013;36(10):1280-3.

35. Maytin M, Epstein LM, John RM. Lead implant duration does not always predict ease of extraction: extraction sheath may be required at < 1 year. Pacing Clin Electrophysiol. 2011;34(12):1615-20.

36. Rodriguez Y, Garisto JD, Carrillo RG. Laser lead extraction in the octogenarian patient. Circ Arrhythm Electrophysiol. 2011;4(5):719-23.

37. Kratz JM, Toole JM. Pacemaker and internal cardioverter defibrillator lead extraction: a safe and effective surgical approach. Ann Thorac Surg. 2010;90(5):1411-7.

38. Gaca JG, Lima B, Milano CA, Lin SS, Davis RD, Lowe JE, et al. Laser-assisted extraction of pacemaker and defibrillator leads: the role of the cardiac surgeon. Ann Thorac Surg. 2009;87(5):1446-50; discussion 50-1.

39. Scott PA, Chow W, Ellis E, Morgan JM, Roberts PR. Extraction of pacemaker and implantable cardioverter defibrillator leads: a single-centre study of electrosurgical and laser extraction. Europace. 2009;11(11):1501-4.

40. Roux JF, Page P, Dubuc M, Thibault B, Guerra PG, Macle L, et al. Laser lead extraction: predictors of success and complications. Pacing Clin Electrophysiol. 2007;30(2):214-20.

41. Kennergren C, Bucknall CA, Butter C, Charles R, Fuhrer J, Grosfeld M, et al. Laser-assisted lead extraction: the European experience. Europace. 2007;9(8):651-6.

42. Moak JP, Freedenberg V, Ramwell C, Skeete A. Effectiveness of excimer laser-assisted pacing and ICD lead extraction in children and young adults. Pacing Clin Electrophysiol. 2006;29(5):461-6.

43. Ghosh N, Yee R, Klein GJ, Quantz M, Novick RJ, Skanes AC, et al. Laser lead extraction: is there a learning curve? Pacing Clin Electrophysiol. 2005;28(3):180-4.

44. Bracke FA, Meijer A, van Gelder LM. Lead extraction for device related infections: a single-centre experience. Europace. 2004;6(3):243-7.

45. Cooper JM, Stephenson EA, Berul CI, Walsh EP, Epstein LM. Implantable cardioverter defibrillator lead complications and laser extraction in children and young adults with congenital heart disease: implications for implantation and management. J Cardiovasc Electrophysiol. 2003;14(4):344-9.

46. Byrd CL, Wilkoff BL, Love CJ, Sellers TD, Reiser C. Clinical study of the laser sheath for lead extraction: the total experience in the United States. Pacing Clin Electrophysiol. 2002;25(5):804-8.

47. Costa R, Martinelli Filho M, Crevelari ES, Stolf NA, Oliveira SA. Laser assisted extraction of pacemaker and implantable defibrillator leads. Arq Bras Cardiol. 2001;77(3):235-42.

48. Gilligan DM, Dan D. Excimer laser for pacemaker and defibrillator lead extraction: techniques and clinical results. Lasers Med Sci. 2001;16(2):113-21.

49. Epstein LM, Byrd CL, Wilkoff BL, Love CJ, Sellers TD, Hayes DL, et al. Initial experience with larger laser sheaths for the removal of transvenous pacemaker and implantable defibrillator leads. Circulation. 1999;100(5):516-25.

50. Kennergren C. First European experience using excimer laser for the extraction of permanent pacemaker leads. Pacing Clin Electrophysiol. 1998;21(1 Pt 2):268-70.

51. Krishnan SC, Epstein LM. Initial experience with a laser sheath to extract chronic transvenous implantable cardioverter-defibrillator leads. Am J Cardiol. 1998;82(10):1293-5, A10.
